# Pathophysiology of Spasticity: Implications for Neurorehabilitation

**DOI:** 10.1155/2014/354906

**Published:** 2014-10-30

**Authors:** Carlo Trompetto, Lucio Marinelli, Laura Mori, Elisa Pelosin, Antonio Currà, Luigi Molfetta, Giovanni Abbruzzese

**Affiliations:** ^1^Department of Neuroscience, Rehabilitation, Ophthalmology, Genetics, Maternal and Child Health, University of Genoa, Largo Daneo 3, 16132 Genoa, Italy; ^2^Department of Medical-Surgical Sciences and Biotechnologies, Sapienza University of Rome, Polo Pontino, Via Firenze, 04019 Terracina, Italy

## Abstract

Spasticity is the velocity-dependent increase in muscle tone due to the exaggeration of stretch reflex. It is only one of the several components of the upper motor neuron syndrome (UMNS). The central lesion causing the UMNS disrupts the balance of supraspinal inhibitory and excitatory inputs directed to the spinal cord, leading to a state of disinhibition of the stretch reflex. However, the delay between the acute neurological insult (trauma or stroke) and the appearance of spasticity argues against it simply being a release phenomenon and suggests some sort of plastic changes, occurring in the spinal cord and also in the brain. An important plastic change in the spinal cord could be the progressive reduction of postactivation depression due to limb immobilization. As well as hyperexcitable stretch reflexes, secondary soft tissue changes in the paretic limbs enhance muscle resistance to passive displacements. Therefore, in patients with UMNS, hypertonia can be divided into two components: hypertonia mediated by the stretch reflex, which corresponds to spasticity, and hypertonia due to soft tissue changes, which is often referred as nonreflex hypertonia or intrinsic hypertonia. Compelling evidences state that limb mobilisation in patients with UMNS is essential to prevent and treat both spasticity and intrinsic hypertonia.

## 1. Introduction

Spasticity is a stretch reflex disorder, manifested clinically as an increase in muscle tone that becomes more apparent with more rapid stretching movement. It is a common consequence of lesions that damage upper motor neurons causing upper motor neuron syndrome (UMNS).

The main objectives of this paper are (1) to describe the clinical features of spasticity as one component of UMNS; (2) to describe the mechanisms of muscle tone in normal subjects; (3) to show that spasticity is due to an exaggeration of stretch reflexes caused by an abnormal processing of sensory inputs in the spinal cord; (4) to show that muscle hypertonia in patients with UMNS is also caused by muscle shortening and fibrosis (intrinsic hypertonia); (5) to show that lesions damaging upper motor neurons disturb the balance of supraspinal inhibitory and excitatory inputs controlling the stretch reflex; (6) to describe changes of stretch reflex excitability in the spinal cord triggered by the upper motor neurons dysfunction; and (7) to underline that limb mobilisation in patients with UMNS is essential to prevent and treat both spasticity and intrinsic hypertonia.

## 2. Definition and Clinical Features

The core feature of spasticity is the exaggeration of stretch reflexes. The result is the velocity-dependent increase in resistance of a passively stretched muscle or muscle group. In 1980, Lance published this frequently cited definition: “Spasticity is a motor disorder characterised by a velocity-dependent increase in tonic stretch reflexes (muscle tone) with exaggerated tendon jerks, resulting from hyperexcitability of the stretch reflex, as one component of the upper motoneuron syndrome” [1]. This definition emphasizes the fact that spasticity is just one component of UMNS.

Besides the dependence from velocity, spasticity is also a length-dependent phenomenon. In the quadriceps, spasticity is greater when the muscle is short than when it is long [2, 3]. This is probably one of the mechanisms underlying the so-called clasp knife phenomenon. Bending the knee, at first (when the muscle is short) a great resistance is met. Then, when the quadriceps lengthens, the resistance suddenly disappears. Another mechanism underlying the clasp knife phenomenon could be the excitation of higher-threshold muscle receptors (groups III and IV) belonging to the flexor reflex afferents [4]. On the contrary, in the flexor muscles of the upper limb [5] and in the ankle extensors (triceps surae) [3], spasticity is greater when the muscle is long.

Spasticity is more often found in the flexor muscles of the upper limb (fingers, wrist, and elbow flexors) and in the extensor muscles of the lower limb (knee and ankle extensors). However, there are several exceptions. For example, we observed patients in whom spasticity is prevalent in extensor muscles of the forearm.

## 3. Stretch Reflex and Muscle Tone in Healthy Subjects 

In healthy subjects, stretch reflexes are mediated by excitatory connections between Ia afferent fibers from muscle spindles and *α*-motoneurons innervating the same muscles from which they arise. Passive stretch of the muscle excites the muscle spindles, leading Ia fibers to discharge and send inputs to the *α*-motoneurons through mainly monosynaptic, but also oligosynaptic pathways. The *α*-motoneurons in turn send an efferent impulse to the muscle, causing it to contract.

Surface EMG recordings in a normal subject at rest clearly show that passive muscle stretches, performed at the velocities used in the clinical practice to assess muscle tone, do not produce any reflex contraction of the stretched muscle. For instance, recording the EMG of elbow flexors during imposed elbow extension, no stretch reflex appears in the biceps when the passive displacement occurs at the velocities usually used during the clinical examination of muscle tone (60°–180° per second). It is only above 200° per second that a stretch reflex can be usually seen. Therefore, stretch reflex is not the cause of the muscle tone in healthy subjects. The muscle tone in healthy subjects is completely due to biomechanical factors [6].

## 4. Muscle Tone in Patients with Spasticity: The Exaggerated Stretch Reflex

Differently from healthy subjects, in patients with spasticity evaluated at rest (completely relaxed), a positive linear correlation between EMG activity of the stretched muscle and stretch velocity was found using a range of displacement velocities similar to that used in the clinical practice to evaluate the muscle tone. When the passive stretch is slow, the stretch reflex tends to be small (low amplitude) and the tone may be perceived relatively normal or just increased. When the muscle is stretched faster, stretch reflex increases and the examiner detects an increase in muscle tone. Therefore, spasticity is due to an exaggerated stretch reflex [6].

**Figure 1:**
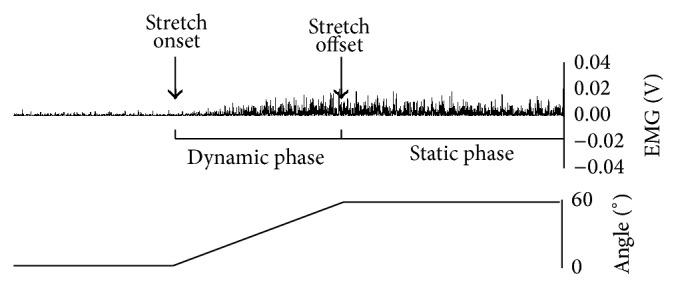
Rectified electromyographic activity recorded from the flexor carpi radialis of a patient with left spastic hemiparesis from right cerebral ischaemic stroke. The muscle has been stretched throughout a range of 60° (dynamic phase) and maintained elongated afterward (static phase). The electromyographic activity not only is present during the dynamic phase, reflecting the typical stretch reflex, but persists also during the static phase.

Although spasticity is velocity-dependent, surface EMG recordings show that in many cases if the stretch is maintained (velocity = 0), the muscle still keeps contracting, at least for a time. So, although spasticity is considered classically dynamic, there is also an isometric tonic muscle contraction after the stretch reflex elicited in a dynamic condition (Figure 1; personal unpublished data).

## 5. Soft Tissue Changes: Intrinsic Hypertonia

Spasticity is responsible for the velocity-dependence of muscle hypertonia in patients with UMNS. However, it must be stressed that in such patients muscle hypertonia is a complex phenomenon, where spasticity represents only one aspect.

Animal studies show that muscle immobilization at short lengths reduces serial sarcomere number [7] and increases the proportion of connective tissue in the muscle [8]. These changes, which emerge very early during immobilisation [9], enhance muscle resistance to passive displacements [10] and increase the resting discharge of muscle spindles and their sensitivity to stretch [11]. It is likely that muscle contracture in patients with UMNS is produced by similar adaptations.

In patients with UMNS, muscle contracture makes a significant contribution to hypertonia [12–14]. Hypertonia in patients with UMNS, therefore, can be divided into two components: hypertonia mediated by the stretch reflex, which corresponds to spasticity, and hypertonia due to muscle contracture, which is often referred as nonreflex hypertonia or intrinsic hypertonia. In contrast to spasticity, in intrinsic hypertonia resistance to passive displacements is not related to the velocity of the movement. However, in a clinical setting it can be difficult to distinguish reflex and nonreflex contributions to muscle hypertonia [15, 16], especially when muscle fibrosis occurs without shortening of the muscle. Biomechanical measures combined with EMG recordings can be helpful in this attempt [17]. It is important to say, however, that the two components of hypertonia are likely to be intimately connected. The reduced muscle extensibility due to muscle contracture might cause “any pulling force to be transmitted more readily to the spindles,” thus increasing spasticity [18].

## 6. The Exaggeration of Stretch Reflex in Patients with Spasticity Is due to an Abnormal Processing of Sensory Inputs in the Spinal Cord

Theoretically, the exaggeration of the stretch reflex in patients with spasticity could be produced by two factors. The first is an increased excitability of muscle spindles. In this case, passive muscle stretch in a patient with spasticity would induce a greater activation of spindle afferents with respect to that induced in a normal subject, of course considering a similar velocity and amplitude of passive displacements. The second factor is an abnormal processing of sensory inputs from muscle spindles in the spinal cord, leading to an excessive reflex activation of *α*-motoneurons.

Classical studies in the decerebrate cat suggest that *γ*-motoneurons hyperactivity and subsequent muscle spindle hyperexcitability have a role in producing hypertonia [19]. On the contrary, studies in humans suggest that fusimotor dysfunction probably contributes little to exaggerated stretch reflex [20]. The commonly accepted view, therefore, is that spasticity is due to an abnormal processing in the spinal cord of a normal input from the spindles.

The velocity-dependence of spasticity can be attributed to the velocity sensitivity of the Ia afferents. However, several studies suggest that II afferent fibers from muscle spindles are also involved in spasticity activating the *α*-motoneurons through an oligosynaptic pathway [21, 22]. It has been suggested that II afferent fibers, which are length-dependent, could be responsible for the muscle contraction in isometric conditions often seen after the dynamic phase of the stretch reflex in patients with spasticity [23].

## 7. Upper Motor Neuron Syndrome: A Complex Picture Where Spasticity Is Only One Component

After a stroke or a trauma damaging upper motor neurons, weakness and loss of dexterity are immediately apparent. Other signs can be hypotonia and loss (or reduction) of deep tendon reflexes. These signs are known as the negative features of the UMNS. Sometime later, other signs appear, characterised by muscle overactivity: spasticity, increased deep tendon reflexes (also called tendon jerks), clonus, extensor spasms, flexor spasms, Babinski sign, positive support reaction, cocontraction, spastic dystonia, and associated reactions. These signs are known as the positive signs of the UMNS. Among them, the only one that tends to appear soon after the lesion, together with the manifestation of the negative signs, is the Babinski sign [24].

The hyperexcitability of the stretch reflex produces spasticity, clonus, and the increase of deep tendon reflexes. Increased excitability of the physiological flexor withdrawal reflex produces flexor spasms of the lower limbs, commonly seen after spinal cord injuries. The release of primitive reflexes (existing at birth but later suppressed during development) is the cause of the Babinski sign and the positive support reaction. The Babinski sign is a cutaneous reflex, while the positive support reaction is a proprioceptive reflex.

On the contrary, cocontraction and associated reactions do not depend on spinal reflexes; therefore, they are efferent phenomena. Also spastic dystonia is thought to depend upon an efferent drive.

Cocontraction is the simultaneous contraction of both the agonist and the antagonist muscles around a joint, for example, the wrist flexors and extensors. In healthy subjects, the voluntary output from the motor cortex activates the motoneurons targeting the agonist muscles and, through the Ia interneurons, inhibits those innervating the antagonist muscles (reciprocal inhibition). In the UMNS, cocontraction is due to the loss of reciprocal inhibition during voluntary command [25]. This is likely to be the most disabling form of muscle overactivity in patients with UMNS, as it hampers generation of force or movement.

Associated reactions are involuntary movements due to the activation of paretic muscles which occur during voluntary activation of unaffected muscles or during involuntary events such as yawning, sneezing, and coughing [26]. An example of associated reaction is the elbow flexion and arm elevation often seen in hemiplegic subjects during walking [23].

Spastic dystonia refers to the tonic contraction of a muscle or a muscle group when the subject is at rest. It can be described as a relative inability to relax muscles [18]. Spastic dystonia can alter resting posture contributing to the hemiplegic posture: the upper limb is flexed and adducted; the lower limb is extended [23]. Although not induced by muscle stretch, spastic dystonia is sensitive to muscle stretch and length. It can be triggered by muscle stretch, even though prolonged stretch can reduce it [18]. The common view is that spastic dystonia is an efferent phenomenon, mediated by an abnormal pattern of supraspinal descending drive [18]. The inability to relax the muscle (i.e., spastic dystonia) is a central feature in spastic patients and is likely to be connected to the prolonged firing of *α*-motoneurons, a well-documented phenomenon in patients with UMNS [27]. We think that this inability to relax the muscle is present not only after a voluntary contraction or after an involuntary event (for instance yawning, sneezing, and coughing), but also after a reflex contraction, possibly having a role in the isometric tonic muscle contraction often seen in spastic patients after the dynamic phase of stretch reflex. We do think that this issue warrants further studies.

## 8. Supraspinal Influences on the Stretch Reflex: Studies in Animals 

In 1946, Magoun and Rhines discovered a powerful inhibitory mechanism in the bulbar reticular formation, in an area immediately behind the pyramids (ventromedial bulbar reticular formation). The stimulation of this area can suppress any type of muscle activity, including stretch reflex activity, both in decerebrate and in intact animals. Studies conducted with the local application of strychnine were the first to show that the ventromedial bulbar reticular formation receives facilitatory influences from the premotor cortex [28]. Accordingly, while the destruction of the primary motor cortex [29] or the interruption of its pyramidal projections in the brain stem [30] caused a flaccid weakness, more extensive cortical lesions, involving premotor and supplementary motor areas, were followed by increased activity of the stretch reflex due to the inhibition of the ventromedial bulbar reticular formation [31]. The inhibitory influences from the bulb are conducted down to the spinal cord by the dorsal reticulospinal tract, which runs very close to the lateral corticospinal tract (pyramidal tract) in the dorsal half of the lateral funiculus [32].

In contrast, the stimulation of the reticular formation of the dorsal brain stem from basal diencephalon to the bulb (dorsal reticular formation) can facilitate or exaggerate any type of muscle activity, including stretch reflex activity [28]. The facilitatory effects, unlike the inhibitory effects of the reticular formation, are not controlled by the motor cortex [33]. The facilitatory influences from the dorsal reticular formation are conducted down to the spinal cord by the medial reticulospinal tract in the anterior funiculus, together with the vestibulospinal tract. The latter, important in the cats as far as the development of hypertonia is concerned, seems to be of declining significance in the primates [34].

**Figure 2:**
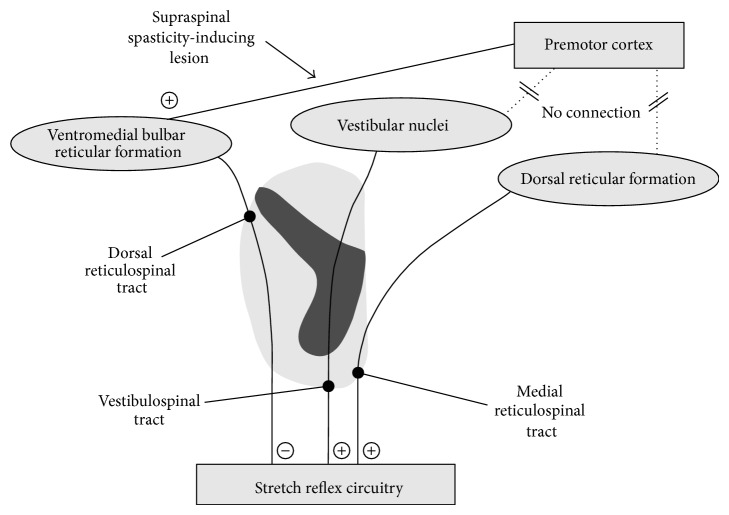
Schematic representation of the descending pathways modulating the stretch reflex circuitry (see text).

In conclusion, studies in animals showed that two major balancing descending systems exist, controlling stretch reflex activity: the inhibitory dorsal reticulospinal tract on one hand and the facilitatory medial reticulospinal and vestibulospinal tract on the other. Only the ventromedial bulbar reticular formation, the origin of the dorsal reticulospinal tract, is under cortical control. The prevalence of the facilitatory system on the inhibitory one leads to the exaggeration of the stretch reflex (Figure 2).

## 9. Supraspinal Influences on the Stretch Reflex: Studies in Humans

These studies provided results in line with those performed in animals. First, spasticity is not related to the pyramidal system. Selective damage to the pyramidal tract at the level of the cerebral peduncle [35] and at the level of the pyramids [36] is not followed by spasticity. Second, spasticity is due to loss or reduction of the inhibitory influences conducted by the dorsal reticulospinal tract. Section of the dorsal half of the lateral funiculus, performed to treat parkinsonism, was followed by spasticity [37]. Third, spasticity is maintained through the facilitatory influences conducted by the medial reticulospinal tract. The vestibulospinal tract plays only a minor role. Section of the vestibulospinal tract in the anterior funiculus of the cord, undertaken by Bucy with the hope of relieving hypertonia, resulted in transient but not permanent reduction in spasticity [38]. In contrast, extensive unilateral or bilateral anterior cordotomy, which is likely to have destroyed both the vestibulospinal tract and the medial reticulospinal tract, was followed by a dramatic reduction of spasticity [39]. Finally, some observations are in line with the finding in animals that the facilitatory corticobulbar system comes from the premotor cortex. Indeed, small capsular lesions in the anterior limb of the internal capsule, where the fibres from the premotor areas are located, tend to be associated with spastic hypertonus, whereas those confined to the posterior limb are not [40].

In conclusion, brain lesions cause spasticity when they disrupt the facilitatory corticobulbar fibers, thus leading to the inhibition of the ventromedial reticular formation, from which the dorsal reticulospinal tract takes its origin. Incomplete spinal cord lesions cause spasticity when they destroy the dorsal reticulospinal tract sparing the medial reticulospinal tract. In the complete spinal cord lesion, both the facilitatory and inhibitory influences on the stretch reflex are lost. As all these tracts inhibit the physiological flexor withdrawal reflex, flexor spasms are predominant [41].

## 10. Changes in Spinal Neuronal Circuitry in Spasticity

Dorsal reticulospinal tract exerts its inhibitory control over the stretch reflex through the activation of inhibitory circuits in the spinal cord. Some inhibitory circuits reduce the excitability of the stretch reflex acting on the membrane of *α*-motoneurons. These circuits are globally defined as postsynaptic inhibitory circuits and their effect is called postsynaptic inhibition. They include disynaptic reciprocal Ia inhibition, Ib inhibition, and recurrent inhibition [42]. Moreover, there is a circuit which reduces the excitability of the stretch reflex acting on the presynaptic terminals of Ia afferents through axoaxonal GABAergic synapses. The activation of this presynaptic inhibitory circuit reduces the release of neurotrasmitters in the synaptic cleft between Ia presynaptic terminals and the membrane of *α*-motoneurons causing presynaptic inhibition [43]. All these postsynaptic and presynaptic circuits can be investigated in humans using neurophysiological techniques based on the H-reflex [44].

Postsynaptic inhibitory circuits have been extensively investigated in patients with spasticity: Ib inhibition [45], disynaptic reciprocal Ia inhibition [46], and recurrent inhibition [47]. In general, all these mechanisms have been found to be decreased in patients with spasticity, supporting the concept that decreased postsynaptic inhibition is involved in the hyperexcitability of the stretch reflex. Also presynaptic inhibition has been found to be depressed in spastic patients with paraplegia [48] and in the upper limb of spastic hemiplegic patients [49].

Besides presynaptic inhibition, postactivation depression is another mechanism reducing the release of neurotransmitters from Ia afferents [50]. Although the molecular mechanisms responsible for postactivation depression are still an open issue [51], it has been shown that postactivation depression reflects an intrinsic neuronal property associated with a decreased probability of transmitter release from the repetitively activated Ia afferents [52]. Therefore, postactivation depression is not mediated by inhibitory spinal circuits and it does not seem to be controlled by descending motor pathways. In comparison to healthy controls, postactivation depression has been found to be lower in patients with spasticity [53]. A positive correlation has been reported between the diminished postactivation depression and the severity of spasticity following stroke [54] and cerebral palsy [55]. Moreover, in subjects with spinal cord injury, postactivation depression is normal in the acute phase and becomes depressed only just before the development of spasticity [56]. Altogether these studies state that postactivation depression plays a pivotal role in the development of spasticity. Compelling evidences in animals [57], healthy subjects [58–60], and spinal cord injured patients [60–63] state that reduction of postactivation depression is mainly caused by limb immobilisation, as that caused by the negative features of the UMN syndrome. We have recently shown that physical exercise can determine a partial normalization of postactivation depression in hemiparetic patients with spasticity following unilateral hemispheric stroke. This partial normalization was accompanied by a decrease of muscle hypertonia in some subjects [64].

## 11. Brain and Spinal Cord Plasticity

In damage from acute events (such as stroke or trauma), the delay between the neurological insult and the appearance of spasticity argues against it simply being a release phenomenon and suggests some sort of plastic changes, occurring in the spinal cord and also in the brain.

In the central nervous system, hypersensitivity of receptors resulting from partial or complete denervation is well documented [65]. The resulting hyperexcitability of the postsynaptic membrane may be caused by the formation of new receptors or by morphological changes in denervated receptors. This phenomenon (denervation supersensitivity) could be implicated in the increased excitability of *α*-motoneurons deprived of their regular descending excitation from the corticospinal pathways. Moreover, *α*-motoneurons after an UMN lesion are known to release growth factors locally [66]. These tend to promote local sprouting from neighbouring interneurons, thus creating conditions for the formation of new abnormal synapses between these interneurons and the somatic membrane of the deprived motor neurons. The new interneuronal endings branch onto the membrane of *α*-motoneurons and occupy the spaces left empty by the missing descending fibers [67], thus leading to the creation of new abnormal reflex pathways [68].

Furthermore, brainstem descending pathways (reticulospinal, vestibulospinal, tectospinal, and rubrospinal tracts) could be increasingly recruited to take over some of the execution of motor command following disruption of the corticospinal pathways. The excitatory connections to spinal motoneurons of these pathways are likely to be less selective than those of the corticospinal tract, leading to muscle overactivity.

Finally, an important mechanism could be the progressive reduction of postactivation depression due to limb immobilization [56, 57].

## 12. Pain and Spasticity

Spasticity can be the direct cause of pain [69]. It has been shown in healthy subjects that lengthening a contracted muscle (eccentric contraction) can cause the disruption of some muscle fibers with the release of substances that may excite the muscle nociceptors [70]. The same process is likely to happen when a spastic muscle is stretched. However, it must be said that all the positive and negative features of UMN syndrome along with soft tissue changes perturb body weight distribution, inducing excessive stress on joint structures and causing pain [23]. Sensory disturbances can also play a role. All these components lead to the pain perceived by the patients with UMNS. The relationship between spasticity and pain is made even more strict by the fact that pain increases spasticity, creating a spiralling course of more pain and disability [71].

## 13. Implications for Neurorehabilitation

This review underlines two aspects of great relevance for rehabilitation. The first point concerns the core feature of spasticity, that is, the exaggeration of stretch reflex. This phenomenon is mediated by several spinal mechanisms ranging from denervation supersensitivity of *α*-motoneurons to the reduced excitability of both postsynaptic and presynaptic inhibitory circuits which control the stretch reflex. These mechanisms, which reflect an aberrant adaptation of the neural circuitry at the spinal level, are actually the result of the lesion of the upper motor neuron. Postactivation depression, conversely, is a phenomenon that controls the excitability of the stretch reflex acting at the spinal level without depending on supraspinal control. It reflects an intrinsic membrane property of Ia afferent fibers, which appears to be independent of the influences exerted by rostral centres. In patients with UMNS, postactivation depression decreases due to limb immobilization, which in turn is caused by weakness and the other negative signs. This is an issue of fundamental importance as passive limb mobilization can restore postactivation depression reducing and even preventing spasticity, as proved by recent findings in humans [62, 64, 70].

The second point is that spasticity is not the only cause of muscle hypertonia in patients with UMNS. In such subjects, muscle immobilization (especially at short lengths) leads to muscle contracture, which makes a significant contribution to hypertonia [12, 13, 18, 64]. Furthermore, muscle fibrosis and the other components of muscle contracture could even increase spasticity through an overactivation of spindle afferents during muscle lengthening [18]. Muscle contractions may be prevented and treated by prolonged muscle stretching [72].

In conclusion, in patients with UMNS, weakness leaves the affected muscles immobilized. The immobilisation in a shortened position leads to muscle contracture, which is the cause of intrinsic hypertonia. At the same time, muscle immobilisation reduces postactivation depression, which is a pivotal mechanism in the development of spasticity. Therefore, in patients with UMNS, mobilization of the affected limbs and the prevention of prolonged shortened position of the affected muscles are probably the most important things to do in order to prevent and treat muscle hypertonia. In this attempt, physiotherapy has an utmost role providing a regular and individualised stretching program, along with the correct positioning of limbs and the applications of splints and casts.
